# Association between Novel Marker (Platelet-Lymphocyte Ratio, Neutrophil-Lymphocyte Ratio, and Delta Neutrophil Index) and Outcomes in Sudden Cardiac Arrest Patients

**DOI:** 10.1155/2021/6650958

**Published:** 2021-03-24

**Authors:** Sang Il Han, Kyoung-Chul Cha, Young Il Roh, Sung Oh Hwang, Woo Jin Jung, Tae Youn Kim

**Affiliations:** Department of Emergency Medicine, Yonsei University Wonju College of Medicine, Wonju, Republic of Korea

## Abstract

**Purpose:**

It is important that clinicians accurately predict the outcome of patients with sudden cardiac arrest (SCA). The complete blood count (CBC) is an easy and inexpensive test that provides information on blood content. Platelet-lymphocyte ratio (PLR), neutrophil-lymphocyte ratio (NLR), and delta neutrophil index (DNI) are relatively novel biomarkers that have been used in the prognosis of various diseases. We aimed to determine the usefulness of PLR, NLR, and DNI in predicting the outcomes of SCA.

**Materials and Methods:**

This retrospective observational study was performed on patients with SCA. Patients who visited the tertiary university hospital from January 2015 to December 2019 were targeted. The inclusion criteria were all nontraumatic adult out-hospital cardiac arrest patients. We analyzed DNI, PLR, and NLR based on the CBC results of all enrolled patients. The exclusion criteria were as follows: no data on laboratory study, traumatic arrest, age < 18 years, and a history of leukemia, myelodysplastic syndrome, and myelofibrosis. The primary outcome was assessed as return of spontaneous circulation (ROSC), the secondary outcome as survival to discharge, and the tertiary outcome as neurological outcome.

**Results:**

From January 1, 2015, to December 31, 2019, 739 patients were enrolled. ROSC was seen in 324 patients, of whom 60 had survival to discharge and 24 had good neurological outcome at the time of discharge (cerebral performance categories (CPCs) 1-2). The PLR of the ROSC group was 42.41 (range: 4.21–508.7), which was higher than that of the No-ROSC group (*p*=0.006). The DNI value of the survival group was 0.00 (range: 0.00–40.9), which was lower than that of the nonsurvival group.

**Conclusions:**

Patients with SCA and subsequent ROSC had higher PLR and NLR, while those with survival to discharge had lower DNI values than those with nonsurvival to discharge (*p*=0.005).

## 1. Introduction

Outcome assessment after acute cardiac arrest is important to determine treatment regimen [1]. Currently, 80% of sudden cardiac arrests (SCAs) have a cardiac origin, such as heart failure or acute coronary syndrome including myocardial infarction. The rest are of noncardiac origin, including respiratory problems, cerebrovascular problems, trauma, and metabolic disorders [2]. In accordance with the advanced cardiopulmonary life support (ACLS) algorithm [3], when cardiac arrest occurs, laboratory study or imaging is conducted to identify reversible causes [4]. Based on laboratory studies, the following Hs and Ts should be treated: hypovolemia, hypoxia, hydrogen ions, and hypo/hyperkalemia [5]. Therefore, laboratory studies are essential for patients with cardiac arrest, and tests that can predict prognosis through serial follow-up have been reported [6–8].

The complete blood count (CBC) is an easy, inexpensive, and routine test that is often used in emergency rooms to provide information about blood content. It can determine white blood cell count, red blood cell count, platelet count, neutrophil count, lymphocyte count, and monocyte count. Neutrophil-to-lymphocyte ratio (NLR) and platelet to lymphocyte ratio (PLR) are used to predict prognosis in various diseases [9–11].

In particular, PLR is associated with malignant tumors, cardiovascular disease, and heart failure [10], probably because inflammatory processes and excessive thrombus activity influence pathogenesis in these diseases [12]. High NLR is associated with most cardiovascular disease outcomes, including acute coronary syndrome, stroke, and composite cardiovascular events [13]. The delta neutrophil index (DNI) has been studied by several research groups as a serum biomarker in infectious and inflammatory conditions [14]. It has implications for sepsis severity and may be valuable to assess prognosis in patients with suspected sepsis [15].

Postcardiac arrest syndrome denotes the biological and clinical manifestations of ischemia-reperfusion injuries triggered by cardiac arrest and return of spontaneous circulation (ROSC) [16]. Systemic ischemia/reperfusion due to cardiac arrest and ROSC is characterized by the release of systemic inflammatory cytokines and generalized activation of leukocytes and endothelial cells [17]. When cardiac arrest occurs, immature neutrophils enter circulation. The elevated immature/total granulocyte ratio is referred to as a left shift [15]. Ischemic damage due to cardiac arrest is associated with more severe apoptosis of T cells and may lead to lower lymphocyte counts [18]. Anticoagulant protein C is consumed rapidly within 2 hours after an arrest, and a short hypocoagulant condition is replaced by a prolonged hypercoagulant condition that causes microcirculatory obstruction, severe lactic acidosis, thrombocytosis, and progressive multiorgan failure [19]. The severity of such disorders varies based on the severity of the ischemic insult, the cause of cardiac arrest, and the patient's state of health before the cardiac arrest [20].

We conducted this study to investigate the significance of PLR, NLR, and DNI in the assessment of outcomes after cardiac arrest.

## 2. Methods

### 2.1. Study Design and Setting

This was a retrospective observational study performed in the emergency department (ED) of a tertiary university hospital visited by 43,000 patients annually. Approximately 200 of those patients present with out-of-hospital cardiac arrest (OHCA). This study was approved by the Institutional Review Board of Wonju Severance Christian Hospital, Yonsei University (IRB No. CR320112).

### 2.2. Participants

**Figure 1:**
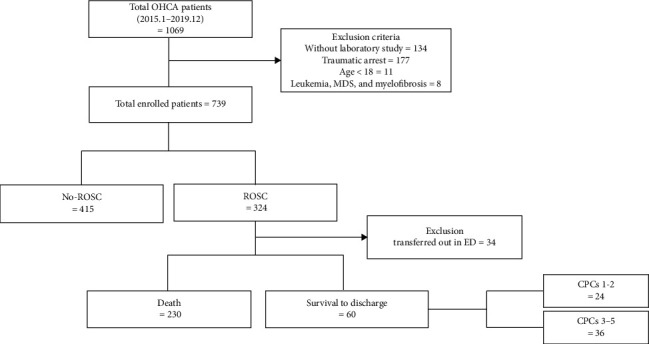
Enrollment chart.

From January 2015 to December 2019, all patients with OHCA who visited the ED were included. The total number of enrolled patients was 1068. All patients underwent treatment according to the current ACLS guidelines [21]. The exclusion criteria were (1) age under 18 years, (2) traumatic cardiac arrest, (3) lack of laboratory study, and (4) insufficient data fidelity in the enrollment chart. We excluded 134 patients with no data on laboratory studies, 177 patients with traumatic cardiac arrest, and 11 patients aged < 18 years. Eight patients were excluded due to a medical history of leukemia, myelodysplastic syndrome, and myelofibrosis. Finally, 739 patients were enrolled in the study (Figure 1).

### 2.3. Study Variables

The following clinical and laboratory parameters were obtained: age, sex, total cardiopulmonary resuscitation (CPR) time, neutrophil count, lymphocyte count, platelet count, PLR, NLR, and DNI. The timing of blood sampling was taken during ACLS. Regarding laboratory data, the serum white blood cell, neutrophil, and lymphocyte counts, the platelet count, and the PLR, NLR, and DNI were checked as part of the CBC (ADVIA 2120i Hematology system; Siemens Healthcare Diagnostics, Deerfield, IL, USA). C-reactive protein (Dimension Vista 1500; Siemens Medical Solutions, USA, Inc., Malvern, PA, USA) was used to predict inflammation and infection in the ED. The DNI is calculated based on the number of immature granulocytes using parameters of ADVIA 2120i, as follows: DNI = [%neutrophil + %eosinophil] − %PMN [11].

### 2.4. Study Endpoints

The primary outcome was the ROSC after ACLS. Secondary outcomes were divided into one patient group, including deaths after ROSC ( ^*∗*^including death in the ED; death in the ICU) and survival to discharge (including  ^*∗*^home discharge and nursing care discharge) among the patients with ROSC. However, patients were excluded when they were referred to other institutions or were hospitalized after ROSC but could not receive postcardiac arrest care because they were transferred to other institutions within the ICU.

Neurological prognosis was considered as a tertiary outcome in patients who survived to discharge. Neurological prognosis was graded using cerebral performance categories (CPCs). This was measured at the time of hospital discharge and was assessed for outcome. Good neurological outcome was defined as CPCs 1-2, and poor neurological outcome was defined as CPCs 3–5 [22].

### 2.5. Statistical Analysis

Continuous variables were expressed as mean ± SD or median (interquartile range), as appropriate, and categorical variables as counts and percentages (%). Equal distribution was tested using the Shapiro-Wilk test. Depending on normality, categorical data were analyzed using Fisher's exact or Pearson's chi-square test, while an unpaired *t*-test or Mann-Whitney *U* test was used to analyze continuous data. Multivariable analysis was performed using a logistic regression model to identify independent predictors of outcome. All *p* values < 0.05 were considered statistically significant, and the analysis was performed using SPSS ver. 23 (IBM, Armonk, NY, USA) and MedCalc Statistical Software version 17.5.3 (MedCalc Software, Ostend, Belgium).

## 3. Results

### 3.1. Baseline Characteristics of the Patients

From January 1, 2015, to December 31, 2019, patients underwent CPR in the hospital's ED. The median age of the enrolled 739 patients was 72 years (range: 19–98 years), and 444 (60.1%) were male. The median total CPR time was 42 minutes (range: 1–194 minutes), the median C-reactive protein level was 0.95 (range: 0.02–45.60), the median DNI was 0% (range: 0%–57.6%), the median neutrophil count was 5.43 × 10^9^/L (range: 0.06 × 10^9^/L–43.67 × 10^9^/L), the median lymphocyte count was 4.53 × 10^9^/L (range: 0.19 × 10^9^/L–19.82 × 10^9^/L), and the median platelet count was 178 × 10^9^/L (range: 5 × 10^9^/L–890 × 10^9^/L). The median PLR was 38.24 (range: 1.94–535.29), while the median NLR was 1.22 (range: 0.04–24.60; Table 1).

### 3.2. Outcome Assessment

Of the 739 patients analyzed during the study period, 324 had ROSC after ACLS and 415 did not. The median age was 68 years (range: 20–96 years) in the ROSC group and 75 years (range: 19–98 years) in the No-ROSC group, showing a significant difference. The total CPR time was 52 minutes (range: 2–194 minutes) in the No-ROSC group and 31 minutes (range: 1–134 minutes) in the ROSC group. DNI showed no significant difference between the two groups. The PLR value of the ROSC group was 42.41 (range: 4.21–508.7), which was significantly higher than that of the No-ROSC group (34.57; range: 1.94–535.29). The NLR in the ROSC group was 1.38 (range: −0.09–24.60), which was significantly higher than that in the No-ROSC group (1.08; range: 0.04–21.48; Table 1).

During the study period, 60 patients with ROSC had survival to discharge, while 230 died in the ED or ICU. Thirty-four patients were transferred from the ED to another institution and were excluded from the secondary outcome assessment. The survival to discharge group was younger than the nonsurvival group. In addition, the survival to discharge group underwent a shorter CPR time.

The C-reactive protein level of the survival to discharge group was 0.40 mg/dL (range: 0.29 mg/dL–21.40 mg/dL), which was lower than that of the nonsurvival group. The DNI of the survival to discharge group was 0.00% (range: 0.0%–40.9%), which was significantly lower than that of the nonsurvival group (0.95%; range: 0.00%–56.20%; Table 1).

The neurological outcomes were evaluated in the 60 patients with survival to discharge. Twenty-four of them had good neurological outcomes, corresponding to CPCs 1-2, while 36 had poor neurological outcomes corresponding to CPCs 3–5. There was a difference in age between the two groups, and there was a significant difference in total CPR time. The PLR was higher in the group with good neurological outcomes than in the group with poor neurological outcomes, but the difference was not significant (Table 1).

### 3.3. Predictive Value of Outcomes in Patients with SCA

Univariate and multivariate logistic regression analyses were performed for the first, second, and tertiary outcomes. The predictors of the primary outcome (ROSC) were age and total CPR time, with odds ratios (ORs) of 0.975 (95% confidence interval (CI): 0.964–0.986) and 0.944 (95% CI: 0.935–0.954; Table 2), respectively.

Age, total CPR time, and DNI were significant predictors of the secondary outcome (survival to discharge). The OR of age was 0.958 (95% CI: 0.938–0.978), while that of DNI was 0.924 (95% CI: 0.859–0.995). The OR of total CPR time was 0.973 (95% CI: 0.954–0.992). Other values did not differ significantly (Table 3).

Univariate and multivariate logistic regression analyses on the tertiary outcome (good neurological outcome) showed that age and total CPR time were significant predictors. The OR of age was 0.950 (95% CI: 0.910–0.991), while that of total CPR time was 0.953 (95% CI: 0.912–0.997; Table 4).

## 4. Discussion

In this study, we investigated the differences in PLR, NLR, and DNI according to outcomes in patients with cardiac arrest. The study was conducted in a relatively large cohort, and the novel marker PLR is meaningful as a study that first suggested an association with cardiac arrest outcome.

According to previous studies, the following biomarkers predict outcome after cardiac arrest and ROSC: interleukin-6, high-sensitivity C-reactive protein, serum neuron-specific enolase concentration, and S-100 protein [23]. In one study, the risk of death in patients with in-hospital cardiac arrest (IHCA) increased when the cut-off value of NLR was higher than 4.5 [24]. In another study, DNI values higher than 3.3% at 24 hours significantly predicted 30-day mortality in patients with pediatric cardiac arrest [25]. In a serial follow-up of patients with OHCA, DNI at 3 days could be used to assess mortality and neurological outcomes [6].

PLR is a relatively novel marker that has not been investigated in patients with cardiac arrest. In cardiovascular disease, cerebral vascular disease, malignancy, and sepsis, lower PLR values have been associated with better outcomes [10, 26, 27]. However, in the present study, the PLR was higher in patients with ROSC, survival to discharge, and good neurological outcomes than in those without. An important pathophysiology underlying the systemic ischemia/reperfusion response is disseminated intravascular coagulation in SCA, which leads to microcirculation disorders and thrombocytopenia [17]. But, multivariate logistic regression showed no significant difference in this regard, but the results were nonetheless contrary to those in other diseases. One animal experiment reported that hypoxia (0.5 atm) reduced the platelet count [28]. However, in other studies, long-term hypoxia reduced platelet count (thrombocytopenia), while short-term hypoxia increased it [29].

Neutrophil survival *in vitro* can be prolonged by hypoxia where the cells' capacity to generate ROS [30]. Areas of infection and inflammation are hypoxic, so neutrophils operating in such environments are subject to functional modulation by hypoxia [31]. Neutrophils react most rapidly at the onset of inflammation, such as in cardiac arrest, and move to the damaged area, causing an acute inflammatory reaction. Therefore, if the initial arrest time is fast, the ROSC rate is also high, and the neutrophil count increases. The faster the arrest detection time from the scene time is, the higher the ROSC ratio is likely to be [32], so the explanatory power of our study can be carried out.

In previous studies, low DNI has been associated with increased survival to discharge [6, 25]. In the present study, the DNI during initial ACLS was lower in the survival to discharge group than in the short-term mortality group, and the survival to discharge group had a lower DNI than the nonsurvival to discharge group. This corroborates previous studies, which have shown an increased risk of infection and organ failure when immunity and coagulation processes are activated after cardiac arrest, ischemia, and reperfusion. Cytokines, soluble receptors, and endotoxins rise in the early stages, causing interaction between neutrophils and endothelium, as well as an increase in immature granulocytes and consequently in DNI [6].

### 4.1. Limitation

In this study, there was likely a selection bias because only single-center, retrospective data were used. In addition, there may have been confounding factors, such as cases in the No-ROSC group in which resuscitation was stopped by a caregiver, as well as medically futile situations such as aortic dissection and terminal cancer. Future studies should take these factors into account when designing their inclusion criteria. In addition, research should be conducted using serial follow-up laboratory data.

## 5. Conclusion

In our cohort of patients with SCA, PLR and NLR were associated with ROSC, but among the three blood cell markers, only DNI was associated with survival to discharge. However, unlike other diseases, PLR and NLR were less valuable as predictors.

## Figures and Tables

**Table 1:** Baseline characteristics of the enrolled patients and comparison of variables by outcomes.

	All patients	Primary outcome			Secondary outcome			Tertiary outcome		
		No-ROSC (*N* = 415)	ROSC (*N* = 324)	*p* value	Death^*∗*^ (*n* = 230)	Survival to discharge (*n* = 60)	*p* value	Poor (*N* = 36)	Good (*N* = 24)	*p* value
Age (years)	72 (19–98)	75 (19–98)	68 (20–96)	0.007	70 (20–96)	57 (22–87)	≤0.001	65.5 (22–87)	49 (29–82)	0.004
Male sex, *n* (%)	444 (60.1%)	254 (61.2%)	190 (58.6%)	0.529	133 (57.8%)	40 (66.7%)	0.273	25 (69.4%)	15 (62.5%)	0.780
Hypertension	325 (44.0%)	188 (45.3%)	137 (42.3%)	0.456	98 (42.6%)	24 (40.0%)	0.828	17 (47.2%)	7 (29.2%)	0.259
Diabetes mellitus	216 (29.2%)	114 (27.5%)	102 (31.5%)	0.268	69 (30.0%)	20 (33.3%)	0.733	13 (36.1%)	7 (29.2%)	0.780
Coronary heart disease	73 (9.9%)	42 (10.1%)	31 (9.6%)	0.900	24 (10.4%)	5 (8.3%)	0.809	2 (5.6%)	3 (12.5%)	0.380
CVA	69 (9.3%)	42 (10.1%)	27 (8.3%)	0.483	20 (8.7%)	4 (6.7%)	0.807	3 (8.3%)	1 (4.2%)	0.643
Previous CPC										
CPCs1-2	681 (92.2%)	386 (93.0%)	295 (91.0%)		209 (90.9%)	58 (96.7%)		34 (94.4%)	24 (100%)	
CPC 3	58 (7.8%)	29 (7.0%)	29 (9.0%)	0.397	21 (9.1%)	2 (3.3%)	0.226	2 (5.6%)	0	0.512
Bystander CPR	406 (54.9%)	219 (52.8%)	187 (57.7%)	0.205	103 (44.8%)	23 (38.3%)	0.452	21 (58.3%)	16 (66.7%)	0.704
Witnessed	392 (53.0%)	197 (47.5%)	195 (60.2%)	≤ 0.001	134 (58.3%)	39 (65.0%)	0.424	22 (61.1%)	17 (70.8%)	0.619
Location										
Public	290 (39.2%)	155 (37.3%)	135 (41.7%)		89 (38.7%)	30 (50.0%)		20 (55.6%)	10 (41.7%)	
Home	449 (60.8%)	260 (62.7%)	189 (58.3%)	0.264	141 (61.3%)	30 (50.0%)	0.150	16 (44.4%)	14 (58.3%)	0.429
Presenting rhythm										
Asystole	446 (60.4%)	296 (71.3%)	150 (46.3%)		112 (48.7%)	19 (31.7%)		16 (44.4%)	3 (12.5%)	
PEA	228 (30.9%)	92 (22.2%)	136 (42.0%)		96 (41.7%)	26 (43.3%)		17 (47.2%)	9 (37.5%)	
Pulseless VT	6 (0.8%)	3 (0.7%)	3 (0.9%)		2 (0.9%)	1 (1.7%)		0	1 (4.2%)	
Ventricular fibrillation	59 (8.0%)	24 (5.8%)	35 (10.8%)	≤0.001	20 (8.7%)	14 (23.3%)	0.007	3 (8.3%)	11 (45.8%)	0.002
Frequency defibrillation	0 (0–38)	1.5 (0–6)	0 (0–38)	0.870	0 (0–38)	0 (0–11)	0.084	0 (0–11)	0 (0–6)	0.003
Total CPR time (min)	42 (1–194)	52 (2–194)	31 (1–134)	≤0.001	32 (1–134)	25.5 (1–72)	≤0.001	29 (2–67)	13.5 (1–72)	0.018
C-reactive protein (mg/dL)^a^	0.95 (0.29–45.6)	0.81 (0.02–45.6)	1.17 (0.29–39.9)	0.091	1.46 (0.29–39.9)	0.40 (0.29–21.4)	≤0.001	1.10 (0.29–21.4)	0.29 (0.29–19.7)	0.007
Delta neutrophil index (%)^b^	0.00 (0.00–57.6)	0.00 (0.00–57.6)	0.45 (0.00–56.2)	0.101	0.95 (0.00–56.2)	0.00 (0.00–40.9)	0.005	0.20 (0.00–16.9)	0.00 (0.00–40.9)	0.189
Neutrophil count (×10^9^/L)	5.43 (0.06–43.67)	4.98 (0.06–43.67)	6.30 (0.12–41.11)	0.001	6.34 (0.12–41.11)	7.58 (0.29–32.81)	0.262	7.48 (0.29–29.64)	7.58 (1.25–32.81)	0.608
Lymphocyte count (×10^9^/L)	4.53 (0.19–19.82)	4.64 (0.19–13.23)	4.34 (0.58–19.82)	0.386	4.21 (0.60–19.82)	5.02 (0.58–13.29)	0.736	7.84 (0.94–13.29)	4.32 (0.58–10.12)	0.163
Platelet count (×10^9^/L)	178 (5–890)	167 (5–643)	188 (11–890)	≤0.001	179 (11–890)	211 (42–431)	0.002	207.5 (110–431)	220.5 (42–377)	0.460
Platelet-lymphocyte ratio	38.24 (1.94–535.29)	34.57 (1.94–535.29)	42.41 (4.21–508.7)	0.006	41.85 (4.21–508.70)	48.83 (7.65–329.67)	0.101	40.53 (14.52–274.34)	60.11 (7.65–329.67)	0.156
Neutrophil-lymphocyte ratio	1.22 (0.04–24.60)	1.08 (0.04–21.48)	1.38 (0.09–24.60)	≤0.001	1.39 (0.09–18.24)	1.69 (0.04–24.6)	0.411	1.43 (0.22–21.15)	2.02 (0.04–24.60)	0.566

CPC, cerebral performance category; CPR, cardiopulmonary resuscitation; PEA, pulseless electrical activity; VT, ventricular tachycardia; ROSC, return of spontaneous circulation.  ^*∗*^Including death in the emergency department and death after hospitalization. ^a^Normal reference range: <0.3. ^b^Normal reference range: <2.6 (%).

**Table 2:** The predictive factors for the primary outcome (ROSC).

Variable	Univariate logistic regression			Multivariate logistic regression		
	OR	95% CI	*p* value	OR	95% CI	*p* value
Age	0.998	0.979–0.997	0.013	0.975	0.964–0.986	≤0.001
Male sex, *n* (%)	0.832	0.668–1.209	0.116	0.926	0.647–1.324	0.673
Total CPR time	0.947	0.938–0.956	≤ 0.001	0.944	0.935–0.954	≤0.001
CRP	1.002	0.984–1.022	0.803			
DNI	1.007	0.989–1.025	0.461			
PLR	1.004	1.001–1.007	0.004	1.003	0.998–1.007	0.223
NLR	1.116	1.050–1.185	≤ 0.001	1.004	0.916–1.100	0.940

CI, confidence interval; CPR, cardiopulmonary resuscitation; DNI, delta neutrophil index; PLR, platelet-lymphocyte ratio; NLR, neutrophil-lymphocyte ratio.

**Table 3:** The predictive factors for the secondary outcome (survival to discharge).

Variable	Univariate logistic regression			Multivariate logistic regression		
	OR	95% CI	*p* value	OR	95% CI	*p* value
Age	0.960	0.941–0.978	≤ 0.001	0.958	0.938–0.978	≤0.001
Male sex, *n* (%)	1.458	0.803–2.650	0.215			
Total CPR time	0.971	0.954–0.988	≤ 0.001	0.973	0.954–0.992	0.006
CRP	0.942	0.894–0.994	0.028	0.984	0.931–1.039	0.561
DNI	0.926	0.869–0.987	0.018	0.924	0.859–0.995	0.035
PLR	1.005	1.001–1.010	0.012	1.006	0.999–1.013	0.081
NLR	1.086	1.010–1.168	0.027	1.014	0.898–1.145	0.821

CI, confidence interval; CPR, cardiopulmonary resuscitation; DNI, delta neutrophil index; PLR, platelet-lymphocyte ratio; NLR, neutrophil-lymphocyte ratio.

**Table 4:** The predictive factors for the tertiary outcome (good neurological outcome).

Variable	Univariate logistic regression			Multivariate logistic regression		
	OR	95% CI	*p* value	OR	95% CI	*p* value
Age	0.951	0.916–0.988	0.011	0.950	0.910–0.991	0.018
Male sex, *n* (%)	0.818	0.247–2.179	0.655			
Total CPR time	0.960	0.926–0.996	0.031	0.953	0.912–0.997	0.035
CRP	0.895	0.776–1.031	0.125	0.887	0.762–1.033	0.123
DNI	1.020	0.933–1.114	0.664			
PLR	1.006	0.999–1.013	0.099	1.004	0.996–1.012	0.318
NLR	1.052	0.950–1.165	0.332			

CI, confidence interval; CPR, cardiopulmonary resuscitation; DNI, delta neutrophil index; PLR, platelet-lymphocyte ratio; NLR, neutrophil-lymphocyte ratio.

## Data Availability

The patient agreed to this data collection and study but did not consent to data sharing.
